# Evolving insights: how DNA repair pathways impact cancer evolution

**DOI:** 10.20892/j.issn.2095-3941.2020.0177

**Published:** 2020-12-15

**Authors:** Jiadong Zhou, Xiao Albert Zhou, Ning Zhang, Jiadong Wang

**Affiliations:** 1 Department of Radiation Medicine, Institute of Systems Biomedicine, School of Basic Medical Sciences, Peking University Health Science Center, Beijing 100191, China; 2 Laboratory of Cancer Cell Biology, Tianjin Medical University Cancer Institute and Hospital, National Clinical Research Center for Cancer, Key Laboratory of Cancer Prevention and Therapy, Tianjin, Tianjin’s Clinical Research Center for Cancer, Tianjin 300060, China; 3 Biomedical Pioneering Innovation Center (BIOPIC) and Translational Cancer Research Center, School of Life Sciences, First Hospital, Peking University, Beijing 100871, China

**Keywords:** DNA repair, cancer evolution, intratumor heterogeneity, genomic instability, drug resistance

## Abstract

Viewing cancer as a large, evolving population of heterogeneous cells is a common perspective. Because genomic instability is one of the fundamental features of cancer, this intrinsic tendency of genomic variation leads to striking intratumor heterogeneity and functions during the process of cancer formation, development, metastasis, and relapse. With the increased mutation rate and abundant diversity of the gene pool, this heterogeneity leads to cancer evolution, which is the major obstacle in the clinical treatment of cancer. Cells rely on the integrity of DNA repair machineries to maintain genomic stability, but these machineries often do not function properly in cancer cells. The deficiency of DNA repair could contribute to the generation of cancer genomic instability, and ultimately promote cancer evolution. With the rapid advance of new technologies, such as single-cell sequencing in recent years, we have the opportunity to better understand the specific processes and mechanisms of cancer evolution, and its relationship with DNA repair. Here, we review recent findings on how DNA repair affects cancer evolution, and discuss how these mechanisms provide the basis for critical clinical challenges and therapeutic applications.

## Introduction

Cancer is a major threat to human health throughout the world. Despite an extraordinary amount of effort, the goal of eradication or even controlling the disease has not been achieved^1^. Cellular complexity and evolutionary characteristics are the main barriers to curing the disease. Cancer clones possess the features of asexual reproduction and are capable of constantly acquiring mutations under the stress of natural or artificial selection, which make them perfectly suitable for the Darwinian adaptive system.

Genomic instability is one of the hallmarks of cancer cells, which contributes to intratumor heterogeneity, and provides the genetic diversity as materials of natural and artificial selection^2^. Genomic instability refers to the increased tendency to acquire genomic alterations, which range from base pair mutations to chromosome aberrations. Genomic instability caused by defective DNA repair is closely associated with tumorigenesis and cancer progression. Gaining insight into how DNA repair pathways participate in tumorigenesis and cancer evolution is crucial for understanding the progression of tumors, for explaining why drug resistance emerges, and for developing more efficient strategies for controlling cancer.

## Cancer evolution

### Concept

Viewing cancer as an evolutionary process is not a new concept. Nowell’s pioneering study in 1976 proposed a hypothesis of tumor evolution, which is driven by stepwise somatic mutations and selective pressure^3^. Because a single neoplastic cell could be the origin of cancer, the latter is often viewed as a clone. Due to genomic instability, numerous individual cells go through diverse mutations, copy number variations, karyotype alterations, and epigenetic alterations, which have distinct effects on the fitness of cancer cells in a heritable way. Under the force of natural or artificial (such as drug treatment) selection and genetic drift, the subclones possessing beneficial alterations expand by delivering advantageous genetic information to their daughter cells^4^. With these features, cancer is now generally considered as a Darwinian evolving process. Subsequent studies in the field of cancer genetics have validated cancer as a genetically and epigenetically evolving system, containing a large pool of heterogeneous cells^5–7^.

Variations among different subclones are mainly observed due to changes of DNA sequences. These changes can be divided into different types including “driver” lesions (which are selectively advantageous) and “passenger” lesions (which are selectively negligible). Driver lesions can be identified by the higher frequency in many neoplasms, when compared with the expected frequencies in the normal background^8,9^, combined with the oncogenic functions of the dysfunctional genes^10,11^. Driver lesions are believed to play an important role in subclone proliferation advantage and tumorigenesis^12^, such as mutations of *TP53* and *BRAF*, which appear in a wide range of cancer types, as well as *BRCA1* and *BRCA2* mutations in breast cancer^13^ and *APC* mutations in adenomatous polyposis^14^. Yet there are challenges in identifying general driver lesions, such as the spatial heterogeneity of sampling^15^ and the heterogeneity among the same types of tumors^16^. Instead, passenger lesions are just “hitchhikers” along with driver lesions, which account for the majority of all cancer mutations^17^. For example, although the truncated mutations of the N-terminus of *APC* are driver lesions, its C-terminal truncation does not influence tumor progression^18^. Studies have shown that the majority of passenger lesions have existed prior to tumor initiation^19,20^, and during cancer progression, new passenger lesions can also be acquired.

During the process of cancer evolution, diverse types and time points of lesion events together with multiple selective pressures shape the trajectory of clonal lineages. Several models depicting dynamic changes of clonal competition over time have been proposed, including linear, branching, neutral, and punctuated evolution^21,22^. The linear evolution model suggests that when a subclone has gained multiple driver mutations, it eliminates almost all previous subclones, resulting in a major dominant subclone. The branching evolution model, instead, supports the idea that multiple subclones from the same ancestor expand simultaneously with increased fitness, presenting a high level of intratumor heterogeneity. The neutral evolution can be considered as an extreme case of the branching evolution, when random mutations with no significant effect accumulate and lead to intratumor heterogeneity. Finally, the punctuated evolution model challenges the classical Darwinian evolution model and suggests that many lesions occur within a short period of time. There are evidences from different systems that support all four models. For example, multi-region sequencing shows that most driver mutations of clear cell renal cell carcinomas are subclonal, supporting the branching evolution model^23^, while chromothripsis in colorectal cancer^24^ and prostate cancer^25^ tends to favor the punctuated model. Furthermore, in support of the neutral model, the multi-region sequencing of a single tumor has revealed extreme genetic diversity without any evidence of positive selection^26^. There are also evidences of the linear model in some early studies^27–29^. However, these results may have limitations in detecting heterogeneous mutations in distinct subclones. Importantly, evidences have shown that the evolution of tumors may mix multiple models during different processes, rather than following a single model^30,31^. Further studies are therefore needed to investigate the dynamics of tumor evolution models, to determine whether selection and fitness play roles in these mechanisms.

### DNA damage and cancer evolution

The molecular mechanisms underlying cancer evolution include changes in genetics and epigenetics of the genome. Many processes can cause alterations of the DNA sequence or epigenetic reprogramming. For example, when DNA replication is stalled by DNA damage on the template strand, translesion synthesis is executed, which is inherently error-prone and can lead to numerous point mutations^32^. Furthermore, a large network of DNA damage-up proteins (DDPs) was discovered recently in *Escherichia coli,* and many of their human homologs were shown to induce endogenous DNA damage when overexpressed^33^. These DDPs participate in different DNA-damage-inducing mechanisms such as reactive oxygen species (ROS) increase by transmembrane transporters and replication stalling by transcription factors. The strong correlation of DDP expression and the mutation load in diverse types of human cancers suggests that DDPs can be important sources of endogenous DNA damage. Epigenetic reprogramming is another remarkable trait of human cancers, which functions in tumor initiation and progression. For example, the promoter hypermethylation of tumor suppressor genes was discovered as an early event in the transformation of human esophageal cancer, and the accumulation of methylation parallels tumor progression^34,35^. In addition, the dynamic regulation of histone acetylation and methylation can also lead to the activation of oncogenes and repression of tumor suppressors, contributing to various stages of cancer evolution^36,37^. Among all the processes causing genetic and epigenetic alterations, the most important one is the defect of DNA repair pathways. In this review, we mainly focus on DNA repair pathways and their relationship with cancer evolution.

**Figure 1:**
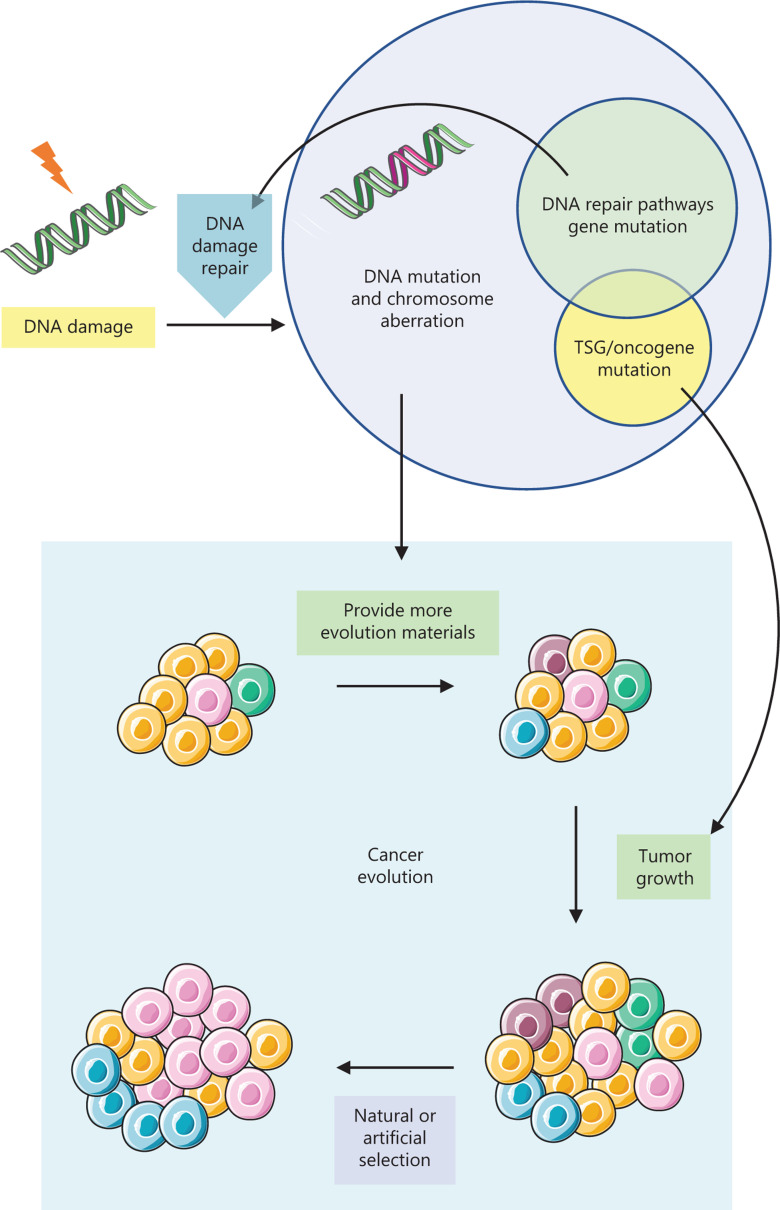
DNA repair pathways influence cancer evolution. When DNA repair pathways are defective, the balance of DNA damage source and repair is shifted, leading to the accumulation of mutations and chromosome aberrations, and an increase of genomic instability. During cancer evolution, moderate genomic instability provides more materials for selection and favors tumor progression. Furthermore, by causing mutations of crucial tumor suppressor genes and oncogenes, genomic instability contributes to the growth advantages of subclones with these driver mutations. Under the natural or artificial (drug treatment) selection, the trajectory of cancer evolution is shaped.

Our genome is constantly assaulted by endogenous and exogenous agents, which can cause numerous DNA lesions and threaten the viability of cells. Some routine physiological processes, such as DNA replication, can lead to spontaneous DNA mutations; and some metabolites such as ROS arising from oxidative respiration can result in endogenous DNA double-strand breaks (DSBs). Furthermore, environmental agents such as ultraviolet light and ionizing radiation can result in exogenous DNA damages^38^. In normal somatic cells, endogenous and exogenous DNA damage can be balanced by efficient repair by multiple DNA repair pathways. However, this damage-repair balance can be shifted by the deficiency of DNA repair pathways, which causes an increase of genomic instability and the accumulation of multiple mutations, finally leading to tumorigenesis (**Figure 1**). Consistently, DNA repair pathway genes also play important roles in species evolution by regulating evolution rates^39^. However, not all cancers harbor an overt level of genomic instability^40,41^, which might be due to a high rate of clonal expansion, and even a relatively normal mutation rate can cause tumorigenesis^42^. The events generated from genomic instability driving cancer initiation can be divided into two classes: small range aberrations such as driver gene mutations, and large range aberrations such as chromosomal rearrangements and the emergence of aneuploid cells^12^. Consistently, a pan-cancer analysis defines two types of tumors^43^. Tumors including kidney clear-cell carcinoma, glioblastoma, acute myeloid leukemia, and colorectal carcinoma, are characterized by mutations (M class), while serous ovarian and breast cancers show characteristics of copy number changes (C class). This difference may be partially due to the defect of different DNA repair pathways, which are discussed in detail in the second part.

During the process of tumor progression and drug treatment, an increased mutation rate raises the diversity of subclones, favoring growth advantages under different selective pressures (**Figure 1**)^44^. However, in this case, more genomic instability is not always better. The level of genomic instability and intratumor heterogeneity vary in different patients and cancer types. Intriguingly, patients with tumors that contain moderate levels of genome instability show the worst prognosis, while tumors with extremely high (for instance, somatic copy number alterations are found in more than 75% of the genome) or too low levels of genomic instability are associated with improved prognosis^45–47^. For example, patients with microsatellite instability (MSI)-positive tumors tend to have favorable prognosis, in which case MSI leads to a dramatic increase of the mutation rate and genomic instability^48^. This phenomenon is called the “trade-off” between tumor genomic instability and tumor fitness, which could influence clinical outcomes^49^. The genomic instability or intratumor heterogeneity is distributed over a wide range. A high level of genomic instability may provide more choices for cancer evolution and tumor progression, but this could also damage the fitness of the tumor, which together may result in a better prognosis. Thus, maintaining a moderate level of genomic instability is critical for tumor cells to survive and compete, because an excessive level of genomic instability can be harmful and reduce cell viability^50,51^. A case in point is that a mouse model with *Cenp-e* deficiency, which leads to increased aneuploidy and chromosome instability, can both promote and inhibit tumorigenesis in different sets of backgrounds^52^. Moreover, using the CIN70 signature to quantify chromosomal complexity and chromosomal instability (CIN), analysis of CIN and patient outcomes in diverse cancers revealed that patients with intermediate CIN had the poorest outcomes, whereas high levels of CIN were correlated with improved outcomes^50^. Therefore, the genomic instability level of tumors has been considered to be maintained in an optimal range, which could facilitate tumor evolution, and cause minimal damage to the tumor itself. Indeed, in many cancer types, mitotic checkpoint genes tend to be highly expressed rather than mutated^53^, and this probably occurs to prevent excessive genomic instability. Furthermore, in colorectal cancers, MSI and aneuploidy are mutually exclusive, suggesting that the combination of MSI and chromosome instability may be lethal to these cancer cells^54^.

This “trade-off” theory could also be applied to clinical drug treatment, because many chemotherapy drugs are aimed to induce more DNA damage and exaggerate the genomic instability of the tumor beyond a certain threshold, leading to the elimination of cancer cells. Cancers with certain defects of DNA repair pathways are specifically sensitive to drugs, which induce corresponding lesions. For example, because DNA adducts caused by cisplatin are known to be detected and repaired by nucleotide excision repair (NER), evidence has linked the expression level of ERCC1 with cisplatin sensitivity in non-small cell lung cancer (NSCLC)^55–57^. Another classic example is the use of inhibitors of poly-ADP-ribose polymerase (PARP) for the treatment of breast and ovarian cancers, which is the first application of the “synthetic lethality” theory (see below). However, these mutagenic therapies could also cause increased mutations, and may result in the promotion of cancer evolution and drug resistance. Strategies are suggested in clinical treatments, including the specific combination therapy of cytotoxic and cytostatic drugs and optimizing the dose of cytotoxic drugs, instead of administering the maximal tolerated dose^58^.

### Clinical significance

The concept of cancer evolution highlights two features of cancer: the intratumor heterogeneity, and the fact that cancer cells undergo a constantly evolving process driven by genomic instability and selective pressure. Studies on premalignant conditions, such as Barrett’s esophagus, have revealed that the level of heterogeneity could be predictive of future neoplasms^59,60^. Furthermore, the levels of intratumor heterogeneity can reflect the potential of tumor progression and provide prognostic information. A study on chronic lymphocytic leukemia showed that subclones with driver mutations, such as mutations of *TP53*, have more potential to expand during chemotherapy, and the presence of subclonal driver mutations serves as an independent risk factor for tumor progression^61^. Furthermore, cancer evolution also poses a huge challenge, in which clinical drug treatments often end with resistance, which will be discussed in detail in the third part.

## DNA repair

The human body possesses sophisticated cancer suppression mechanisms to eliminate or postpone cancer evolution. DNA repair pathways serve as guardians of genomic stability. Thus, it is not surprising that defects of DNA repair pathways are common in various cancers. In normal cells, genomic stability is guarded by an intricate machinery of DNA repair. For different types of DNA damages, there are corresponding repair pathways to efficiently fix them. Defects in these pathways cause diverse and detrimental consequences, and many of them may lead to the generation of cancer (**Table 1**). As mentioned above, the deficiencies of different DNA repair pathways are associated with the features of distinct cancer types. Many of the DNA repair pathway deficiencies leave idiosyncratic patterns of mutations in cancer genomes, termed mutation signatures, which are found in specific types of cancers^62^. For example, breast and ovarian cancers frequently possess mutations of *BRCA1* or *BRCA2*, which are vital genes of homologous recombination (HR), and these cancers often show the mutation signature with a distinctive pattern of substitutions and deletions^63^. Similarly, nucleotide excision repair deficiency is highly associated with dinucleotide mutations, which have been found predominantly in skin cancers. Distinct DNA damage pathways are therefore associated with specific cancer types, and may be involved in multiple processes of cancer evolution. Here, we review several important DNA repair pathways and their impact on cancer evolution by exploring their roles in the generation and progression of cancers.

**Table 1:** DNA repair pathways and their association with cancer evolution

	DNA damage source	Cancer predispose and related disease	Common mutant genes in cancer	Association with cancer evolution when defective
Mismatch repair	DNA polymerization errors	CRC, Lynch syndrome	*MLH1, MSH2, MSH6, PSM2*	Mutator phenotype, adaptive mutability
Nucleotide excision repair	UV, bulky chemical adducts, ROS	Xeroderma pigmentosum, Cockayne syndrome	*XPA, ERCC3 (XPB), XPC, ERCC2 (XPD), DDB2 (XPE), CSA, CSB*	Generating mutations from transcription-associated lesions
Base excision repair	Spontaneous decay of DNA, radiation, cytostatic drugs	CRC, breast cancer, lung cancer	*XRCC1*, *POLB*	Generating mutations, mutator phenotype, and promoting cancer initiation
Double-strand break repair	ROS, DNA replication, IR, radiomimetic chemicals	Breast cancer, ovarian cancer, prostate cancer	*BRCA1, BRCA2, PALB2, MRE11*	Generating mutations and chromosome aberrations, chromothripsis, adaptive mutability
Interstrand crosslink repair	Aldehydes, platinum compounds	Fanconi anemia, breast cancer, ovarian cancer	*BRCA1, BRCA2, FANCM*	Generating mutations and chromosome aberrations from replication associated lesions, tissue specificity

### Mismatch repair

The mismatch repair (MMR) system is critical for maintaining genomic stability, which is highly conserved from prokaryotes to eukaryotes. Its primary function is to eliminate mutational intermediates generated by DNA polymerization errors at the post-replicative level, helping to ensure the high fidelity of DNA replication^64^. MMR in mammalian cells is complex and contains multiple steps. First, mismatched DNA is recognized by either MutSα (MSH2-MSH6) or MutSβ (MSH2-MSH3). Second, MutLα (MLH1-PMS2) is recruited to mismatch sites^65^.Then, PCNA is loaded and activates PMS2 to exert its endonuclease activity by direct interaction^66,67^. Third, during the excision of the mismatched DNA, PCNA and MutLα are required for 3′nick-directed excision, while 5′nick-directed excision is readily carried out by EXO1, which is a 5′→3′ exonuclease and interacts with MSH2 and MLH1^68–72^. RPA is also involved in MMR, which helps to protect the ssDNA generated by the exonuclease, and terminate the excision^73^. Finally, the excised gaps are fixed by DNA polymerase δ and LIG1^74,75^.

Mismatch repair deficiency can cause the expansion and contraction of repetitive tracts of DNA, namely MSI, which leads to an increase of frameshift mutations. MSI is clinically associated with 10%–15% of colorectal, ovarian, endometrial, and gastric cancers, and germline mutation of MMR genes (MLH1, MSH2, MSH6, and PSM2) causes Lynch syndrome, which shows the characteristic molecular changes of MSI and is the most common form of hereditary colorectal cancer (CRC)^76,77^. Moreover, MMR-deficiency has a strong correlation with tumor recurrence and poor patient prognosis following chemotherapy of breast cancer^78^. Importantly, although MMR pathway defect is closely correlated to cancer, MMR deficiency does not by itself lead to cancer. Instead, it causes detrimental mutations in crucial genes, which might contribute to tumor progression, resulting in a “mutator phenotype”^79^. Indeed, in MMR-deficient tumors but not in MMR-proficient tumors, frameshift mutations in mononucleotide tracts have been identified in tumor suppressors and DNA repair genes such as *BRCA1*, *BRCA2* and *BLM*^80,81^, and tumor progression genes such as *BAX*^82^ and *TGFB2*^83^. Evidence has shown that in MMR-deficient cells, an elevated mutation rate could be observed in key growth regulatory genes, which may provide the subclone a growth advantage against selective pressure within a heterogeneous tumor^84^. In addition, MSI in non-transcribed regions can also have a potential tumorigenic effect. For example, a primary cancer series revealed that the expression of a key factor of DSB repair, MRE11, is frequently impaired in MMR-deficient primary colorectal cancers (83.7%), but not in MMR-proficient ones, due to the aberrant splicing caused by the T_11_ repeat instability within the *MRE11* intron 4^85,86^. Therefore, although the hypermutation caused by MSI can also increase the frequency of lethal mutations^87^, MMR deficiency contributes to greater possibilities of driver mutations and epithelial mesenchymal transition (EMT), an increased level of cancer cell genomic instability, and intratumor heterogeneity, ultimately promoting tumorigenesis and favoring cancer evolution^88^.

MMS deficiency and MSI define a unique subset of cancers and have been used as diagnostic tools^89^. In clinical trials, tumors with MMS deficiency or MSI showed great responses to immunotherapy^90^. Furthermore, many studies have shown that MSI is associated with improved prognosis in colorectal and endometrial cancers^91,92^, while CIN, another hallmark of these cancers, is linked to a poor prognosis^50,93^. However, a recent study argued that the association between MSI and a favorable prognosis could be rather due to the lack of aneuploidy of the MSI tumors than the effect of MSI itself^54^. Researchers have shown that there is a clear inverse correlation between MSI and chromosome arm aneuploidy (which refers to the imbalance of chromosome arms) in gastrointestinal and endometrial tumors, suggesting that MSI and aneuploidy are mutually exclusive within these tumors, which leads to an alternative route of cancer evolution with improved prognosis. Further efforts should be directed toward solving questions such as whether combined MSI and aneuploidy could be lethal to tumor cells and may create a potentially new target therapy for aneuploid tumors.

Intriguingly, the MMR pathway, as well as the HR pathway (reviewed below) could directly participate in the response of cancer cells to drug treatment, helping to acquire higher levels of genomic instability and favoring cancer evolution. A recent study demonstrated that EGFR/BRAF inhibition could downregulate MMR genes, and increase mutability and MSI^94^. This research provides evidence for the adaptive mutability of tumor cells, which could evade therapeutic pressure by transiently enhancing mutability, reminding us to pay attention to this novel mechanism of drug resistance and to develop drugs capable of reducing the generation of new mutations.

### Nucleotide excision repair (NER)

The mammalian NER pathway recognizes and repairs a wide range of structurally unrelated DNA lesions, including cyclobutene-pyrimidine dimers and 6-4 pyrimidine-pyrimidone photoproducts induced by ultraviolet (UV) light, bulky chemical adducts, and ROS-generated cyclopurines^95^. There are two subpathways of NER, the global genome NER (GG-NER) and the transcription-coupled NER (TC-NER).

The GG-NER probes the genome for helix-distorting lesions, which are caused by disturbed base pairing. The helix-distorting lesions can be directly recognized by XPC-RAD23B^96^. TFIIH is then recruited to the lesion site through direct interaction with XPC-RAD23B^97^. TC-NER is instead activated by the stalled RNA polymerase II (RNA Pol II) when there is a lesion in the template strand. When the transcription is arrested, the blocked RNA Pol II and elongation complex recruit CSA (ERCC8) and CSB (ERCC6). TFIIH is then recruited, and TC-NER converges with GG-NER^98^. TFIIH opens the double-strand helix around the lesion using its helicase activity. XPA, which can bind single-strand nucleotides, recruits XPF-ERCC1 heterodimer, which excises the lesion using its endonuclease activity^99,100^. The generated gap is filled by DNA Pol δ, DNA Pol κ, or DNA Pol ɛ. Finally, LIG1, or LIG3 is responsible for the nick ligation, finishing the NER reaction^101,102^.

The deficiency of NER can result in a spectral distribution of phenotypes from neurodevelopment defects without cancer predisposition to normal development, but with an extreme predisposition to cancer, due to NER pathway’s diverse functions and subpathways. NER defects cause xeroderma pigmentosum and Cockayne syndrome. Patients of xeroderma pigmentosum show a very strong cancer predisposition due to the genome-wide accumulation of lesions bypassed by error-prone translesion DNA polymerases, thereby increasing mutagenesis. Xeroderma pigmentosum patients have a strong predisposition of many types of skin cancer, mainly squamous cell carcinomas and basal cell carcinomas^103^. Evidence has shown that this predisposition is caused by the inability to repair base damage, especially from UV radiation, leading to somatic mutation and eventually to neoplastic transformation^104^. Moreover, NER deficiency is associated with a higher risk of bladder cancer^105^ and breast cancer^106^, suggesting the broad influence of the loss of NER. Importantly, human adult stem cells lacking NER protein XPC exhibit a three-fold increase in acquired base substitutions, and a 17-fold increase in double base substitutions per week, indicating that cancer cells possessing NER mutations may have more potential to accumulate mutations of a particular signature, driving their evolution^106^.

#### Transcription-associated DNA damage

Because the TC-NER process is so closely coupled with transcription, it is not surprising that it has a strong link with transcription-associated DNA damage. R-loops, which are formed during transcription, are important sources of endogenous DNA damage and genomic instability^107^. A previous study has shown that NER nucleases, XPF and XPG, have the capability to process R-loops *in vitro*^108^, indicating the possible involvement of NER in R-loop cleavage. Later research revealed that TC-NER, rather than GG-NER, is responsible for breaks at R-loop sites^109^. Furthermore, the TC-NER pathway may play a routine role in the clearance of R-loops, because of the additive effect of R-loop accumulation being noticed when XPG and AQR are co-depleted. However, questions remain unanswered on the topic of NER-dependent R-loop processing. For example, TC-NER appears to produce DSBs rather than assumed single-strand breaks (SSBs) at R-loops. Moreover, a recent study suggested an alternative mechanism in which Rad52 is recruited to DSBs in an R-loop-dependent manner during active transcription, promoting XPG, rather than XPF-, to mediate transcription- associated HR^110^. Thus, there is controversy regarding whether the cleavage of R-loops by XPG is directly linked with NER. Although R-loops cause transcription stress and may lead to subsequent replication stalling, TC-NER-dependent R-loop-induced DSBs are also direct dangers to genome stability^111^. NER deficiency causes hypermutations, which is favorable for tumorigenesis and cancer evolution, and may decrease the level of damage caused by R-loop cleavage. However, it remains largely unknown whether this processing is actually a beneficial event, and whether it plays a role in tumorigenesis.

In addition to R-loops, NER plays a role in other forms of transcription-associated mutagenesis. During transcription, transient, site-specific DSBs are often spontaneously induced by DNA topoisomerases on promoters or gene bodies. XPG and XPF may be involved in transcription-associated DSB repair through certain subpathways of HR and through non- homologous end joining (NHEJ)^112,113^. In addition, TC-NER participates in the transcription-coupled repair of oxidative DNA lesions^114^, and some features of the Cockayne syndrome are believed to generate from the deficiency of transcription- associated oxidative DNA repair^115^. Importantly, a recent study reported an important source of transcription-related endogenous mutations caused by interfering with NER^116^. Using excision-repair sequencing data, the study found that regions with active transcription factor binding sites showed a highly increased rate of somatic mutations in melanomas, due to a decrease of NER machinery accessibility to the DNA in these regions, although it remains to be determined whether this mechanism is specific to NER.

Because of the multifunction of NER proteins and the complexity of the pathway cross-talking and intersecting with other DNA repair pathways, the role of the NER pathway in affecting genomic instability is significant, but still not completely understood.

### Base excision repair

Oxidation, deamination, and alkylation can cause DNA base lesions, which are repaired by base excision repair (BER). Spontaneous DNA decay and environmental factors such as radiation and cytostatic drug treatment are both sources of DNA base lesions, and deficiency of BER leads to genomic instability and the predisposition to different human cancers.

BER begins with altered base recognition and removal by DNA glycosylase, leaving an abasic site (AP-site), which is subsequently processed by AP-endonuclease 1 (APE1). APE1 can cleave the phosphodiester bond 5′ to the AP-site, leading to a SSB, which is repaired by the DNA polymerase β-XRCC1-LIG3 complex, completing the so-called short patch BER. In the long patch BER, the 5′-sugar phosphate is resistant to pol β-dependent cleavage, thus it needs to be processed in a FEN1- and PCNA-dependent manner, and the nick is finally sealed by LIG1^117^.

Many components of BER are associated with a high risk of cancer. A polymorphic variant of XRCC1 was found to increase the risk of lung cancer and breast cancer among Asians^118,119^. Furthermore, about 30% of all human cancers express DNA polymerase β variant proteins, indicating the strong link of DNA polymerase β mutations with cancer susceptibility. For example, the K289M mutation of DNA polymerase β has been shown to have lower fidelity for DNA synthesis, thus possibly causing a mutator phenotype and an increase in genomic instability^120^.

Because alkylating agents used in chemotherapy achieve cytotoxicity by inducing base damages, BER proteins can be exploited as prognostic factors of the patients’ response to chemotherapy, and modulating BER, such as APE1 inhibitors, has the potential to improve outcomes of melanoma and glioma patients^121^. Moreover, BER proteins have shown the potential of clinical application as targets and predictors in pancreatic cancer^122^. For example, XRCC1 polymorphism can be a potential predictive marker for responses to platinum-based therapy in NSCLC patients^123^. Furthermore, similar to NER, BER can also be discerned by specific mutation signatures. Defective *MUTYH*, a glycosylase in BER, has been associated with enrichment of transversion mutations and colorectal cancer^124^. Deficiency of another BER gene, *NTHL1*, is characterized by C>T mutations and predisposes to colorectal cancers and polyposis, suggesting that the BER pathway contributes to tumor initiation in these cancers^125^.

### Double-strand break repair

#### NHEJ or HR

DSBs are serious threats to the integrity and stability of the genome, which could cause point mutations, chromosomal rearrangements and cell death. DSBs can be generated by endogenous factors such as ROS and DNA replication, or exogenous agents such as ionizing radiation or radiomimetic chemicals. In normal human cells, there are well-evolved machineries to repair DSBs, including two pathways called HR and NHEJ. In the G1 phase, NHEJ is the dominant pathway to repair DSBs, which directly ligates two ends of the DSB and is often associated with small insertions and deletions^126^. In the S/G2 phase, with the sister chromatid available, cells tend to use HR to carry out DSB repair by using sister DNA as a template in an error-free manner^127^.

During classic NHEJ, the ring-like KU70-KU80 heterodimer initially binds to the double-strand DNA ends and subsequently recruits and activates DNA-PKcs^128^. DNA-PKcs can tether two broken DNA ends in close proximity to facilitate end-joining, and can also recruit downstream end-processing factors such as Artemis^129^, and rejoining executors, LIG4 and XRCC4^130,131^. After DNA end processing, LIG4 performs its ligation activity and results in an intact DNA double strand. Importantly, a key factor of NHEJ, 53BP1, acts as a crucial regulator of NHEJ and HR pathway choice, which favors NHEJ over HR during the G1 phase^132,133^. 53BP1 is recruited to the DSB sites by histone modifications including ubiquitination and methylation^134–137^. Two effectors of 53BP1 are PTIP and RIF1. The nuclease Artemis is the major downstream effector of PTIP^129^. Artemis can slightly trim the broken ends with its endonuclease activity, creating substrates suitable for end-joining and preventing extensive DNA end resection-channeled HR. RIF1 acts downstream of 53BP1 and functions in DNA end protection^138^. In addition, recent studies have identified the Shieldin complex as a key regulator, which is downstream of RIF1 to restrain DNA end resection^139–142^. Thus, 53BP1 plays an important role in the HR/NHEJ pathway choice by antagonizing DNA end resection mediated by BRCA1 (see below), generating a mutual balance model of 53BP1-BRCA1, which has great implications regarding embryonic lethality of BRCA1 deficiency and clinical drug resistance of breast cancer^143^.

HR is initiated by DNA end resection, generating 3′single-strand DNA overhangs at the DSB sites. This process requires highly regulated steps including short-range resection executed by CtIP and the MRE11-RAD50-NBS1 (MRN) complex, followed by the long-range resection carried out by EXO1 or DNA2. During end resection, BRCA1 is required to counteract 53BP1 by directly interacting with CtIP and stabilizing the MRN-CtIP complex^144^. Moreover, another binding partner of BRCA1, PALB2, has been shown to promote BRCA2 recruitment to the DSB site^145,146^. BRCA2 is essential to stabilize RAD51, which replaces RPA at the 3**′** ssDNA overhang and forms a RAD51-ssDNA nucleofilament^147^. The RAD51-ssDNA nucleofilament then searches for a homologous sequence and generates D-loops by DNA-strand invasion, thereby facilitating high fidelity repair through DNA synthesis using the homologous DNA as a template^148^. Finally, D-loops are resolved to either crossover or non-crossover products, depending on the types of HR^149^.

In principle, the dysfunction of DSB repair leads to an increase of genome instability, which in turn causes higher mutation rates of other genes, and ultimately results in cellular transformation and tumorigenesis. Inactivation of DSB repair genes is closely associated with cancer. An elevated mutation load of DSB repair genes is responsible for various hereditary cancers. One of the most well-known examples is that, a large fraction of hereditary breast cancer, ovarian cancer, and prostate cancer, which are accounted for by *BRCA1* and *BRCA2* mutations, which are pivotal genes in HR^150^. In tumor cells with HR deficiency such as BRCA1 mutations, DSBs that would be repaired by HR in normal conditions are instead shunted to the NHEJ pathway, which aberrantly joins DNA ends, leading to massive chromosomal rearrangements^151^ and causing carcinogenesis or even embryonic lethality. This point of view was validated by numerous studies^132,152,153^. For example, loss of 53BP1 alleviates the phenotypic abnormalities of Brca1-mutant mouse embryonic fibroblasts and embryonic stem cells, including spontaneous chromosomal instability and drug sensitivity. More importantly, compared to adult Brca1^Δ11^/^Δ11^ p53^+^/^-^ mice, mammary carcinogenesis is almost fully prevented in adult Brca1^Δ11^/^Δ11^ 53BP1^-^/^-^ mice, suggesting that 53BP1 loss can rescue the genomic stability and prevent tumorigenesis resulting from BRCA1 deficiency^132^.

#### Massive disaster: chromothripsis

Notably, DSB repair failures can create not only point mutations but also large chromosomal alterations. Strikingly, recent studies revealed that many cancer cells harbor tens to hundreds of clustered chromosome rearrangements, with evidence indicating that these detrimental events can be generated by a single catastrophic event, termed chromothripsis^24,154,155^. Chromothripsis leads to the massive disruption of tumor suppressor genes and the formation of oncogene fusion products, suggesting the possibility of a punctuated equilibrium mechanism of cancer evolution, in which several tumor-promoting changes occur in rare and rapid events during tumor progression^156^. Thus, chromothripsis may represent a key point of cancer evolution, and indicates that there is no return in the development of drug resistance^157^.

An initial error in mitosis is believed to produce micronucleus^158,159^. The spatially isolated chromosomes in micronucleus can easily acquire DNA damage in the form of DSBs during S phase from slowed or completely stalled DNA synthesis, and these unrepaired DSBs and/or unresolved replication forks could be catastrophic during mitosis^160,161^, forming chromatin fragments passively distributed in the main nucleus of daughter cells. The DSB ends of these fragments are reassembled predominantly by NHEJ, for depleting or inhibiting LIG4 or DNA-PKcs preventing the repair of micronuclei-derived fragments^162^. Sequence analysis of the breakpoint junctions has revealed that most of them lack significant homology or microhomology, which is consistent with the features of NHEJ^156^, although some doubtful points need to be resolved. For example, micronucleus chromatin DSBs may not be able to trigger the intact DNA repair pathway, and micronuclei with γH2AX often fails to recruit detectable levels of 53BP1^163^.

In summary, DSB repair deficiency can lead to the mutation and dysfunction of tumor suppressor genes and fusion or amplification of oncogenes, triggering tumorigenesis. During the progress of cancer, loss of DSB repair causes an increase in the occurrence of mutations and chromosomal rearrangements, which provide more material for natural or drug selection, and fuels tumor progression.

### Interstrand crosslink repair

DNA interstrand crosslinks (ICLs) can result from endogenous metabolism products such as aldehydes generated from alcohol and dietary fat. Notably, many chemotherapeutic agents such as platinum compounds can cause ICLs. ICLs are serious threats to cells, as they are able to block essential cellular processes such as replication and transcription. In human cells, ICLs are repaired by the Fanconi anemia (FA) pathway, which is also known as the BRCA pathway, containing 19 components (FANCA to FANCT). Some FA proteins are also involved in HR, such as BRCA1, BRCA2, and RAD51, as processing of ICLs induces the generation of DSBs. ICL repair requires various steps and the cooperation of the FA, NER and HR pathways^164^.

ICLs cause replication fork stalling, and the convergence of replication forks is required to unload the CMG complex, favoring subsequent ICL repair^165^. BRCA1 (FANCS) is also involved in this process^166^. The ICL repair begins with FANCM recognizing and binding to ICL, forming a platform for the Fanconi anemia core complex, containing 14 proteins. The Fanconi anemia core complex acts as a large ubiquitin ligase for the heterodimer FANCD2-FANCI. Mono-ubiquitinated FANCD2 facilitates the recruitment of several factors including SLX4 (FANCP), FAN1, and ERCC4 (also known as XPF, which also functions in the NER pathway)-ERCC1 to promote DNA incision and unhook the ICLs^167–170^. Then, lesion bypass is carried out by low fidelity translesion synthesis polymerases, DNA polymerase ζ, or REV1^171,172^, followed by ligation. Finally, DSBs caused by DNA incisions are repaired by HR, with the BRCA2 (FANCD1)-PALB2 (FANCN)-RAD51 (FANCR) axis playing a key role^173^.

The FA pathway is closely correlated to DNA replication, and multiple components participate in the stabilizing of stalled replication forks^174^. Deficiency of FANCA, FANCD2, BRCA1, or BRCA2 leads to increased instability of stalled replication forks, which results from excessive nucleolytic degradation^175,176^. Indeed, the FANCD2-I heterodimer is phosphorylated by ATM and ATR, which are two vital kinases in DNA repair signaling, under replication stress induced by hydroxyurea treatment^177^, and it is co-localized with stalled replication forks^178^. Similarly, RAD51 and BRCA1 are also recruited to stalled replication forks, which are independent of HR in ICL repair^179^. Moreover, FANCD2 cooperates with BLM and facilitates the recruitment of FAN1, which mediates the fork restart^180,181^. In addition, FANCJ, in cooperation with FANCD2 and BRCA2, is required for replication fork recovery^182^. These collectively indicate that the regulation of stalled replication forks is also a major function of FA pathway proteins, and the FA pathway plays an important role in preserving genomic stability and preventing tumorigenesis.

Dysfunctional FA pathway leads to the accumulation of mutations, which combined with multiple constant endogenous and exogenous DNA insults, may result in uncontrolled cell proliferation and ultimately malignant transformation. Because the FA pathway is closely related to DNA replication, its defect can cause replication fork collapse and chromosome breakage, generating high level genomic instability. In FA patients’ cells, chromosome breakage can be induced by crosslinking agents, and it has been used in the FA screening and diagnosis^183^. The FA pathway is also involved in cell division, which is another factor of cancer evolution in addition to mutation accumulation, because only divisions result in the expansion of cells carrying mutations. Furthermore, abnormal cell division could itself generate aberrant daughter cells and favor malignant transformation. Multiple FA proteins, such as FANCD2 and BRCA2, can localize to centrosomes to ensure the normal function of the mitotic apparatus^184^. Impaired spindle assembly checkpoint and an aberrant number of centrosomes have been found in FA patient cells, which can lead to incorrect chromosome segregation, increased genomic instability, and the risk of cancer^185^. Individuals with the FA pathway defect exhibit genomic instability, bone marrow failure, and a strong predisposition to various types of cancers^186^. In addition to BRCA1 and BRCA2 mentioned above, other FA genes such as *FANCN*^145^ and *FANCJ* (BRIP1)^187,188^ have also been implicated in increased risks of breast and ovarian cancers. However, a recent cohort study shows that there is no evidence that truncated mutations of BRIP1 are associated with increased risk of breast cancer, which suggests that large systematic studies are required to refine our estimates of the cancer predisposition of rare FA gene mutations carriers^189^. Moreover, evidence has shown that *FANCT* (UBE2T)^190^ and *FANCM*^191^ gene aberrations are also linked to a higher risk of breast cancer, although further larger-scale studies are needed.

#### Oncogene-induced replication stress

DSBs are the most detrimental damage form, and they can activate the DNA damage checkpoint, which leads to apoptosis and senescence, serving as a barrier to tumor progression. In response to DSB, ATM phosphorylates and activates p53 and CHK2, leading to cell cycle arrest, which is pivotal for cells to repair DSBs and prevent genomic instability^192^. It is well-known that the DNA damage checkpoint gene, *TP53*, is frequently mutated in human cancers. In the view of cancer evolution, the selective pressure, which results in *TP53* mutations, can be explained by the oncogene-induced replication stress model^193–195^.

Mutations of oncogenes such as *KRAS, CDKN2A, EGFR,* and *MYC* promote cell proliferation. This high proliferation state leads to the dysregulation of replication forks, including aberrant origin firing, decreased or increased firing, depleted dNTP pool, and increased transcription-replication conflicts^196^. Overall, these factors can lead to replication fork stalling and collapse, followed by activation of DNA damage checkpoints and p53-dependent apoptosis or senescence. During replication stress, some specific genomic sites, called common fragile sites, are preferentially affected, which exacerbate genomic instability. Eventually, checkpoint genes such as *TP53* are targeted, and the mutations of *TP53* allow cells to escape the fate of apoptosis and senescence, and facilitate tumor progression. In addition to the fact that DNA damage response precedes p53 mutations and gross genomic instability, the mutation patterns of *TP53*, *ATM* and another tumor suppressor, *CDKN2A,* in high-throughput sequencing studies also convincingly support the oncogene-induced replication stress model^197^.

This revolutionary model explains that premalignant lesions already presents DNA damage caused by oncogenes, which trigger proliferation, emphasizing that replication stress is the fundamental origin of DNA damage during cancer evolution.

## Clinical implications of DNA repair involved in cancer evolution

### Tissue specificity of BRCA-associated cancers

The tumorigenesis of germline BRCA1 and BRCA2 deficiencies has an intriguing breast and ovarian specificity, suggesting that in these tissues, BRCA1 or BRCA2 mutant cells are involved in distinct processes of somatic evolution. For BRCA1 mutation carriers, there is a predominant predisposition of breast and ovarian cancer, while BRCA2 mutation carriers also show a significantly higher risk of pancreatic, prostate, and other types of cancers in addition to breast and ovarian cancers^198,199^. More intriguingly, in addition to the tissue preference, BRCA1 and BRCA2 mutation carriers tend to develop different kinds of breast cancer. BRCA1 mutation breast cancers are predominantly triple negative/basal-like, which means these cancers lack the expression of human epidermal growth factor receptor 2, estrogen receptor (ER), and progesterone receptor (PR). In contrast, BRCA2 mutation cancers are mainly luminal type and are ER positive^200–203^.

The mechanism of this tissue specificity is a long-standing question in the field of DNA repair and remains largely elusive. Several models have been brought up, involving multiple functions of BRCA genes including transcription regulation^204,205^. Here, we mainly focused on the DNA-damage-repair-related functions of BRCA1 and BRCA2.

Studies in both mouse models and human tissues have shown that BRCA1-associated breast cancer may arise from aberrant luminal epithelial progenitor cells of the mammary gland^206,207^, which may endow the cancer triple-negative molecular signature. Mice harboring the BRCA1 mutation in luminal epithelial progenitors develop tumors that resemble human BRCA1-associated breast cancer, while BRCA1-mutated basal cells develop distinct tumors. Moreover, luminal progenitors from patients harboring BRCA1 mutations give rise to tumors with distinctive basal differentiation^208^. Along with the DNA repair functions of BRCA1, increased genomic instability has been shown in the mammary epithelial cells of BRCA1 mutation carriers^209^, suggesting the preexisting differences of the DNA damage response at least partially contribute to the tissue preference of BRCA1-associated cancers^210^.

Recent studies have explained why luminal progenitors are more prone to DNA damage and rely on normal functions of BRCA1, showing that the expression of the RANK/RANKL pathway engaging in progesterone signaling, distinguishes the two populations of luminal progenitors in preneoplastic mammary tissues from BRCA1 mutation carriers. High expression of RANK is associated with a high rate of proliferation, making more spontaneous DNA damages in RANK+ luminal progenitors, resulting in the higher sensitivity of replication inhibitors and more dependency on normal BRCA1^211^. This finding highlights the value of RANKL inhibitors as a new preventive therapeutic strategy for BRCA1 mutation carriers. Similarly, the progesterone-associated proliferation of a subpopulation of luminal progenitors leads to persistent activation of the NF-κB pathway and a higher level of DNA damage^212^. Furthermore, evidence has shown that the inadequate repair of ICL damage is associated with transdifferentiation of mammary epithelial cells, resulting in a mesenchymal-like phenotype^213,214^. Using *in vivo* single cell analysis, studies have shown that a defect of the FA pathway caused by BRCA1 depletion can lead to spontaneous DNA damage and can elicit an aberrant differentiation state of mammary epithelial cells, which gives rise to the evolution from normal luminal epithelial cells towards tumor cells through the EMT^213^. The results of these studies link the DNA repair function and maintenance of proper mammary cell differentiation function of BRCA1, highlighting the central role of BRCA1 during breast cancer evolution.

A major risk factor of breast cancer is high levels of estrogen^215^, which indicates that estrogen-induced DNA damage may be a key reason of BRCA-associated tumor preference in breast and ovarian cancers. Metabolites of estrogen can cause ROS-related DSBs specifically in S phase of ER-negative breast cells, indicating that estrogen can induce increased replication stress and subsequent genomic instability^216^. These studies raise the possibility that estrogen may make cells more sensitive to BRCA1 deficiency and contribute to the tissue specificity of BRCA1-associated cancer^217^. However, further studies are needed to determine whether this mechanism could also apply to BRCA2-associated cancer. In turn, in ER-positive breast cells, estrogen can trigger downstream transcription, generating R-loops at E2-responsive genes and replication-dependent DSBs^218^, which may cause these cells to be more vulnerable and rely on BRCA1/BRCA2. Further studies are needed to determine whether the global genomic instability features of BRCA1/BRCA2-associated cancer are related to estrogen-induced DNA damage.

### Developing resistance

**Figure 2:**
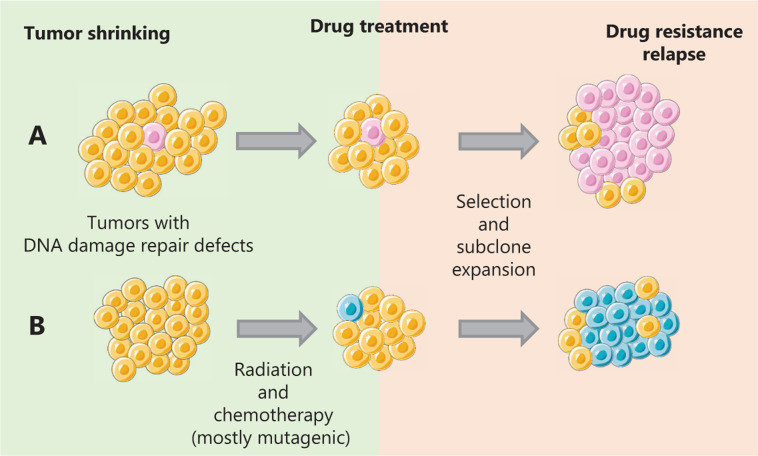
Two distinct evolution models of drug resistance of cancer. (A) The drug resistant subclones reside within the tumor before the drug treatment. Under a selection effect, these subclones eventually expand and become the dominant subclones, leading to drug resistance and tumor relapse. (B) Mutagenic treatments such as alkylating agents can result in an increase of genomic instability and the generation of new driver genes, creating new drug resistant subclones.

Targeted therapeutic drugs have produced good clinical curative effects. However, drug resistance has emerged as one of the biggest problems. Unfortunately, drug resistance is largely due to the intrinsic propensity of cancer itself. Recently, single-cell sequencing has depicted the evolutionary trajectories of cancer development and has been used in studying tumor samples from patients and diverse cancer models^219,220^. Based on these and former studies, there are two main models of drug resistance in the perspective of cancer evolution (**Figure 2**). The evolution could be the result of pre-existing drug resistant tumor subclones, which are rare at first but expand and become the dominant subclone of cancer; alternatively, the emergence of new resistant subclones due to genomic instability and certain drug treatments can also cause resistance^221^. These two mechanisms are discussed below.

Recent studies have revealed that the pre-existence of resistant subclones is the major cause of drug resistance in cancer cell lines^222,223^. Numerous studies have shown that drug-resistant subclones reside within the tumor before treatment of colorectal cancer^224^, ovarian cancer^47^, and melanoma^225^ and prostate cancer^226^. Functioning as a selective pressure, drug treatment causes these subclones with beneficial genomic features to expand, causing resistant subclones to gradually become the dominant groups of cancer, and resulting in drug resistance and cancer relapse. Prolonged BRAF inhibitor treatment for melanoma xenografts carrying BRAF activation mutations leads to resistance. However, the resistant tumor cells exhibit a dependency on the BRAF inhibitor, indicating that new therapeutic strategies can be exploited by targeting this specific vulnerability^227^. Similarly, a recent study based on single cell sequencing of chemotherapy resistant human triple negative breast tumor samples revealed that most mutations in resistant tumors originate from pre-existing genomic alterations^219^. Moreover, this adaptive resistance may be due to the reactivation of the targeted pathways. For example, cisplatin resistance in ovarian cancer is found to be associated with the re-expression of FANCF^228^ and the reverse mutation of functional BRCA1 or BRCA2^229,230^. Similarly, platinum chemotherapies can cause reverse drug resistance through the hypermethylation of mismatch repair genes^231,232^. To solve this problem, DNA-demethylating agents have been combined with platinum to prevent hypermethylation-caused resistance^233^, although it is not the ultimate solution.

Cancer therapies usually kill cancer cells more efficiently than normal cells, and this specificity is key to eliminating cancer without causing significant side effects. Most cancer cells are characterized by more rapid proliferation. Therefore, cell division can be targeted to prevent cancer progression. DNA-damaging drugs are widely used to achieve this goal by causing cell cycle arrest or cell death. However, because these drugs cause more DNA damage, they inevitably induce more mutations and chromosome aberrations during treatment, fueling cancer evolution and eventually causing the emergence of resistant subclones^234,235^. Chemotherapy drugs impact DNA replication, DNA repair, and cell division, generating mutations and chromosome instability, inevitably promoting drug resistance by selecting for the resistant subclones with growth advantages. Studies of acute myeloid leukemia and glioma have shown that the cytotoxic effect of alkylating agents may lead to an increase of the mutation rate and promote the generation of new driver mutations^236,237^. This effect could be intensified, especially in cases with certain defect DNA repair pathways. Studies have shown that in gliomas with *MSH6* inactivation, alkylating agents seem to induce a hypermutation phenotype, which promotes neoplastic progression rather than tumor cell death^238^. Similarly, the cytotoxicity caused by radiotherapy is particularly due to DSBs, and the high efficiency of DSB repair is associated with radioresistance in many cases^239,240^. Furthermore, the mutagenic feature of radiotherapy cannot only contribute to resistance but also can cause radiation-induced neoplasms^239–243^. Notably, in addition to these well-known mutagenic treatments, targeted drugs such as cetuximab could cause adaptive mutability of colorectal cancers by downregulating DNA repair gene expression and aggravating genomic instability^94^.

For cancers with defects in DNA repair pathways, cancer cells often rely on the compensating repair pathway to maintain a suitable level of genomic instability. Thus, targeting such compensating pathways using the concept of synthetic lethality is an effective way to treat DNA repair-defective tumors^244^. One notable example is the PARP inhibitor used in the treatment of inherited breast and ovarian cancer patients with *BRCA1* or *BRCA2* gene mutations. Unfortunately, PARP inhibitor therapy is also hampered by emerging resistance. The mechanism of PARPi resistance has been investigated extensively^245^. Studies have shown that besides general resistance mechanisms for most drugs including the upregulation of drug efflux and target loss^246^, restoration of competent HR by reactivation of functional BRCA proteins^247^, loss of NHEJ by 53BP1 or Shieldin complex defects^140,248^, and restoration of replication fork protection^249^ are also important mechanisms of PARPi resistance.

There are several ways to avoid or overcome the possible acquired resistance of PARPi and other drugs. First, tumors rewiring their DNA repair pathway to gain resistance may have new vulnerabilities to other targeted therapies. For example, BRCA1-mutated tumor cells, which also harbor 53BP1 inactivations, show resistance to PARPi, while this phenotype depends on ATM activity, which makes these cancer cells specifically vulnerable to ATM inhibition^132^. Similarly, loss of NHEJ leads to hypersensitivity to ionizing radiation^139^. Second, combination therapies can be used to sensitize the small subpopulations in cancer, which leads to clonal evolution. For example, the synthetic lethal approach could be used in BRCA-deficient breast cancer, which may be eradicated by certain alkylators or high dose chemotherapy^250^. Finally, it is important to monitor the dynamics of tumor genomic alterations during the process of treatment. Analysis of circulating tumor DNA (ctDNA) is very helpful in providing a broader picture of clonal evolution^251,252^. The ctDNA can reveal somatic mutations acquired through therapy and can mitigate tumor sampling bias, making it a powerful tool to delineate evolutionary trajectories of individual cancer and to guide therapeutic interventions.

### Stress-induced drug tolerance

The traditional view of evolution biology states that mutations are random, constant, and gradual, and the generation of variation and natural selection are two independent processes. In the field of cancer evolution, a similar view has been challenged by discoveries of the reversible drug-tolerant epigenetic state and stress-induced adaptive mutability of cancer cells under treatment.

Transient drug resistance caused by nongenetic alterations has been reported in many studies^253,254^. In a ground-breaking study, during drug treatment, a small population of cells with a drug-tolerant phenotype was detected. These cells can maintain viability under near-extinction conditions with >100-fold reduced drug sensitivity^255^. Intriguingly, this phenotype is transiently acquired and KDM5A is responsible for the chromatin alterations in the drug-tolerant state, and altering histone acetylation can prevent the development of acquired drug resistance. Consistent with clinical reports of the success of a “drug-holiday,” this study also suggests that chromatin-modifying agents can be utilized to improve clinical benefits.

Stress-induced mutagenesis has long been recognized in *Escherichia coli,* and the similarity between cancer cells and asexually reproducing prokaryotes has suggested that the same mechanism may exist in human cancers to develop drug resistance^256^. Recently, a vital discovery of adaptive mutability in response to targeted therapy in human cancer evolution has provided a new perspective to investigate how DNA repair functions during tumor progression^94^. In this study, Russo et al. provided evidence that cancer cells possess a similar mechanism to generate intratumor heterogeneity, which enables cancer adaptation during drug treatment. Human CRC cells treated with EGFR or BRAF inhibitors showed a transient dysfunction of MMR and HR repair systems and a corresponding increase of DNA damage and MSI. More importantly, this mutagenesis ceased after drug removal. Furthermore, this study suggested that DNA repair deficiencies underlying drug treatment may create new therapeutic vulnerabilities for the development of combined therapies^257^. Because CRC cells are HR deficient during treatment, it is worth exploring whether PARPi could show a better efficiency to kill these cancer cells. Moreover, a recent study showed that the Werner syndrome ATP-dependent helicase (WRN) may play a similar role as a synthetically lethal target in MMR deficient cancers^258^, thus WRN inhibition may be an alternative way to target these cancer cells. Similarly, platinum such as oxaliplatin, a DSB-inducing drug, may be a greater threat to these cancer cells. Subsequently, a new study suggested that stress-induced mutagenesis is a prevalent feature of human cancers, and a genome-wide screen identified Serine/threonine-protein kinase mTOR as a stress-sensing capacitor regulating accurate DNA replication and repair^259^. These studies demonstrated that DNA repair is at the core of cancer evolution and can be regulated under different environmental conditions.

## Conclusions

Intratumor heterogeneity and cancer evolution make cancer one of the biggest challenges of modern medicine, and the most insidious killers of humans. As genomic technologies advance rapidly in recent decades, our understanding of how cancer evolution is driven and shaped becomes enhanced. In this perspective, many basic problems still need to be solved. It would be significant to distinguish the driver mutations and passenger mutations and trace the pivotal event, which makes normal cells turn into cancer cells. DNA repair pathways play crucial roles in maintaining genomic stability, and how cancer cells hijack DNA repair pathways to keep intratumor instability in a moderate range to fuel cancer evolution is largely unknown. Furthermore, the prevalence of adaptive mutability should be more extensively assessed in the future. By understanding these mechanisms, we can gain more insight into the characteristics of diverse tumor types, which can be coupled to treatment strategies and benefit individual patients. Analyses of mutation signatures in cancer genomes have shown the great potential of tumor diagnostics and treatment guidance. We can also explore how to develop future therapeutic strategies to slow down the pace of cancer evolution and to avoid or overcome drug resistance, by targeting not only DNA repair pathways, but also other important characteristics possessed by cancer cells during evolution, such as alterations of transcription, replication, and epigenetics. For example, to combat the reversible drug tolerant condition in osimertinib treatment of NSCLC, HDACi shows promising clinical application, by targeting chromatin state, which has been mentioned above. In addition, combined therapy can be exploited to overcome drug resistance. For example, mutagenic therapies such as radiotherapy can induce the production of neoantigens, which can act as an *in situ* vaccine. Thus, patients may benefit from the combination of chemotherapy/radiotherapy and immune checkpoint blockade therapy.

The characteristics of intratumor heterogeneity and cancer evolution pose a big challenge for the development of targeted therapies and more effective drugs. Increasing our understanding of the mechanisms driving genomic instability and cancer evolution is key to developing therapeutic approaches to accurately limit cancer diversity, evolution, and drug resistance in individual patients.
